# Effects of Two Protein Hydrolysates Obtained From Chickpea (*Cicer arietinum* L.) and *Spirulina platensis* on *Zea mays* (L.) Plants

**DOI:** 10.3389/fpls.2019.00954

**Published:** 2019-07-25

**Authors:** Andrea Ertani, Serenella Nardi, Ornella Francioso, Santiago Sanchez-Cortes, Michele Di Foggia, Michela Schiavon

**Affiliations:** ^1^Dipartimento di Agronomia, Animali, Alimenti, Risorse Naturali e Ambiente (DAFNAE), Università di Padova, Padua, Italy; ^2^Dipartimento di Scienze e Tecnologie Agro-Alimentari, Università di Bologna, Bologna, Italy; ^3^Instituto de Estructura de la Materia, IEM-CSIC, Madrid, Spain; ^4^Dipartimento di Scienze Biomediche e Neuromotorie, Bologna, Italy

**Keywords:** hormone-like activity, ELISA, peroxidase and esterase enzymes, FT-IR, SERS, *Spirulina platensis*, *Cicer arietinum* L.

## Abstract

Two liquid protein hydrolysates obtained from chickpea (*Cicer arietinum* L.) (CA) and *Spirulina platensis* (SP) were analyzed via FT-IR and SERS spectroscopy. Their hormone-like activities and contents in indole-3-acetic acid (IAA), isopentenyladenosine (IPA), nitrogen (N), carbon (C), sulfur (S), phenols, amino acids, and reducing sugars were determined. CA and SP showed different chemical compositions in N, C, sugars, amino acid, and TP contents, which were generally higher in CA. The two products exhibited (IAA)-like and gibberellin (GA)-like activities and contained the hormones IAA and IPA. Specifically, CA held higher (∼3.6 fold) IAA-like activity than SP, while its GA-like activity was comparable to SP. The content in IAA was similar between hydrolysates, but CA contained ∼6 fold more IPA. CA and SP were further supplied at two different dosages (0.1 and 1 mL L^–1^) for 2 days to maize (*Zea mays* L.) plants grown in hydroponics. They positively influenced plant growth and accumulation of N-compounds (proteins, chlorophylls and phenols), with a more pronounced effect observed in plants treated with CA. Furthermore, they increased the activity of two enzymes, i.e., peroxidase and esterase, which are established markers for plant growth, differentiation and organogenesis-related processes. Peroxidase activity in particular, was enhanced by ∼1.6 and ∼2.3 fold in leaves and roots of CA-treated plants, respectively. Greater accumulation of macro (Ca, Mg, and K) and micro (Cu, Zn) elements was also evident in plants supplied with these products. In conclusion, our data indicate that both CA and SP exert positive effects in maize plants. However, CA appeared to be more efficient than SP to improve plant nutrition and growth parameters in some respects, likely by virtue of its higher content in phytochemicals (hormones, phenols, amino acids, reducing sugars) that may act as signaling molecules, and more pronounced IAA-like activity.

## Introduction

In the last decades, feeding on high quality vegetables has gained increasing attention by consumers and has pushed crop research toward designing sustainable strategies aimed to improve food nutritional valuable traits. One of these strategies involves the use of biostimulants, which consist of mixtures of inorganic/organic compounds and/or microorganisms that can be applied during crop cultivation to ameliorate yields, especially under sub-optimal conditions (Matteo et al., 2013; Nardi et al., 2016; Backer et al., 2017; Posmyk and Szafrańska, 2017; De Pascale et al., 2018). Biostimulants at very low dosages stimulate plant nutrition via up-regulation of nutrient transporters (Ertani et al., 2015, 2016, 2017b). They can also mitigate the adverse effects of abiotic stress via induction of stress-related genes and antioxidant molecules (Ertani et al., 2013c, 2017a; Posmyk and Szafrańska, 2017), and enhance the plant content in beneficial phytochemicals.

Commercial biostimulants comprise three main categories, humic substances, seaweed extracts and protein hydrolysates (Schiavon et al., 2008), whose activity has been frequently ascribed to their content in signaling molecules, such as small peptides, amino acids, hormones and phenolic compounds (Schiavon et al., 2008; Ertani et al., 2011a,b, 2013a, 2015, 2018; Aremu et al., 2012; Colla et al., 2014). However, given the complexity and potential interactions of their different constituents, the mechanism through which biostimulants prompt physiological responses in plants is often hard to dissect.

Humic substances (HS) are macromolecules derived from a complex and heterogeneous aggregation process of small-size molecules, which are the main components of soil organic carbon. They are able to promote plant growth via increased soil nutrient bioavailability (Clapp et al., 2001; Chen et al., 2004; Vaccaro et al., 2015; Nardi et al., 2016) and activation of signaling transduction pathways implied in plant development processes (Nardi et al., 2009; Quilty and Cattle, 2011). HS contain the hormones auxins (Schiavon et al., 2010) and cytokinins (Ertani et al., 2013b; Pizzeghello et al., 2013), which can influence plant cell metabolism (Piccolo et al., 1992; Nardi et al., 2000, 2017a, 2017b).

Seaweed extracts have been widely employed as biofertilizers over centuries, and more recently as biostimulants, because of their positive effects on plant performance, yield and resistance to stress (Khan et al., 2009). Brown seaweeds (e.g., *Ascophyllum nodosum, Macrocystis pyrifera*, and *Durvillea potatorum*) in particular, are commonly used in food processing, industrial and horticulture applications (Khan et al., 2009). They promote plant growth, seed germination rate, chlorophyll synthesis, fruit quality, earlier flowering and fructification, production of secondary roots (Khan et al., 2009; Roussos et al., 2009; Ali et al., 2015; Satish et al., 2015; Goñi et al., 2016; Ertani et al., 2017a). They can also induce resistance to pathogens and abiotic stress in plants (Joubert and Lefranc, 2008; Sharma et al., 2014; Santaniello et al., 2017).

Protein hydrolysates (PHs) are another important group of biostimulants (Nardi et al., 2016; Colla et al., 2017). They can be produced through chemical (acid and alkaline hydrolysis) and/or enzymatic hydrolysis of animal and plant raw materials (Ertani et al., 2009), in liquid or soluble forms, and are supplied to plants via either foliar spraying or direct application to roots of plants growing in hydroponics (Colla and Rouphael, 2015). Like HS and seaweed extracts, protein hydrolysates are used to increase the productivity and quality of a wide range of crops. They can alleviate osmotic and oxidative stress in plants caused by drought, heavy metals and salinity (Chen and Murata, 2008; Apone et al., 2010; Ertani et al., 2013a; Rouphael et al., 2017). The mode of action of PHs is not completely understood, but they contain amino acids, small peptides and hormones (e.g., auxins, triacontanol) that may play a role as signaling molecules in several cellular metabolic and growth processes (Schiavon et al., 2008; Ertani et al., 2013b). An alfa alfa protein hydrolysate has been reported to up-regulate the expression of genes and increase the activity of enzymes involved in N assimilation and C primary metabolism (Schiavon et al., 2008; Ertani et al., 2009), and enhance the secondary metabolism that drives the synthesis of phenolic compounds (Ertani et al., 2011b, 2013c, 2017a). Consistently with these studies, a protein-based product in solid form has been recently shown to promote maize plant growth, protein synthesis and polyphenol accumulation (Ertani et al., 2018).

One of the main interests of the current research on biostimulants is developing new products with very efficient stimulatory properties on plant productivity (Rouphael and Colla, 2018). In this respect, the characterization of biostimulants appears to be a powerful tool in predicting their efficacy and helps in understanding how they act in the plant whether their chemical properties and functional groups are crossed with the physiological effects induced.

In this study we evaluated the effects of two different protein hydrolysates obtained from the legume chickpea (*Cicer arietinum* L.) and from the microalga *Spirulina platensis* on some nutritional and growth parameters of maize plants grown in hydroponics. Before testing these products on plants, we first determined their chemical properties (e.g., hormone-like activity, and content in main elements, organic nitrogen, hormones, individual and total phenols, amino acids, and soluble sugars). The protein hydrolysates were also characterized using vibrational spectroscopic techniques, in particular Fourier Transformation Infrared (FT-IR) spectroscopy and Surface Enhanced Raman Scattering (SERS) technique that allows the detection of strong Raman signals from analytes in solution. The novelty stays in that while the IR spectroscopy is a well-established technique in the characterization of different matrices including protein hydrolysates (Ertani et al., 2018), SERS has been used for the first time in this research field. Indeed, SERS technique detects analytes in extremely diluted solution in presence of noble metals nanoparticles and has been only recently applied in studies of plant materials (Palanco et al., 2016) and algal biofilms (Ramya et al., 2010).

## Materials and Methods

### Chemical and Spectroscopic Characterization of Protein Hydrolysates

The protein hydrolysates, CA from chickpea (*C. arietinum* L.) seeds and SP from *S. platensis*, were obtained by fully controlled enzymatic hydrolysis (FCEH®) performed by ILSA S.p.A (Arzignano, VI, Italy). First, the raw material was dispersed in water inside stored bio-reactors equipped with temperature, weight and pH control. Then, a pool of selected proteolytic and cellulolytic enzymes was used, and the mixtures obtained were kept for 12 h under continuous shaking, at 55–60°C, i.e., the most suitable temperature for the bio-catalytic activity of the enzymes. When the enzymatic reaction was ended, the liquid suspensions were subjected to centrifugation, clarification and filtration. In this last phase, deactivation of the enzymatic pool also took place simultaneously. After a further filtration of the products, stabilized, clear and free of sediments, they were finally stored.

Total carbon (TC), nitrogen (TN) and sulfur (S) contents were quantified using a dry combustion procedure inside an elemental analyzer (vario MACRO CNS, Hanau, Germany). Organic nitrogen (NO) was determined via Kjeldahl method reported in the official methods of Italian soil chemical analysis (Violante, 2000). Total phenol compounds (TP) were determined according to the method by Arnaldos et al. (2001). CA and SP (1 mL) protein hydrolysates were added with pure methanol (1:1, v/v) for 30 min and centrifuged at 5,000 *g* for 30 min at 4°C. 1 mL of 2% (w/v) Na_2_CO_3_ and 75 μL of Folin-Ciocalteau reagent (Sigma-Aldrich) were added to 100 μL of phenolic extract. After 15 min of incubation at 25°C in the dark, the absorbance at λ = 725 nm was measured. Gallic acid was used as standard according to Meenakshi et al. (2009).

For reducing sugars determination, a sample of each product was added with 0.1 N H_2_SO_4_. Samples were incubated in a heating block for 40 min at 60°C and then centrifuged at 6,000 *g* for 10 min at 4°C. After filtration (0.2 μm, Membra-Fil^®^ Whatman Brand, Whatman, Milan, Italy), the supernatants were analyzed by HPLC (Perkin Elmer 410, Perkin Elmer, Waltham, MA, United States). Soluble sugars were separated through a Biorad Aminex 87 C column (300 × 7.8 mm; Bio-Rad Laboratories, Inc., Hercules, CA, United States) using H_2_O as eluent at a flow rate of 0.6 mL min^–1^.

For free amino acids quantification, the method of Seebauer et al. (2004) was used. Fifty milligram of homogenous dry powder of each PH was extracted for 1 h at room temperature with 1.5 mL of a 5% (w/v) TCA solution. The samples were clarified by centrifugation, and 1.5 mL of the supernatants analyzed. The amino acid analysis was performed through a pre-column OPA derivatization of the sample followed by reverse phase separation, using an Agilent 1100 HPLC (Agilent Technologies, Palo Alto, CA, United States) equipped with a thermo-controlled auto-sampler, fluorescence detector and an Agilent HP Chemstation for data elaboration. The chromatographic conditions were described previously by Henderson et al. (2000).

For the analysis of individual phenolic acids, 1 mL of either CA or SP products was added with deionized water (1:2) and shaken for 1 h at 25°C. The samples were then centrifuged at 13,000 *g* for 40 min and filtered (0.22 lm; Membra-Fil^®^ Whatman Brand, Whatman, Milano, Italy). Phenols were separated using HPLC 2700 (Thermo Finnigan, San Jose, CA, United States) coupled with an 1806 UV/Vis (Thermo Finnigan, San Jose, CA, United States) detector. The column TM-LC 18 (Supelcosil) was equipped with pre-column TM-LC 18 (Pelliguard, Supelco). Elution was performed with a flow rate of 1.2 mL min^–1^ using a mixture of water/*n*-butanol/acetic acid (80.5:18:1.5 v/v) as mobile phase. The volume of sample injected was 20 μL. Detection of single phenols was performed at λ = 275 nm and the identification of compounds was achieved by comparing their retention time values with those of corresponding standards (chlorogenic acid, caffeic acid, *p*-cumaric acid and ferulic acid). The calibration curve and quantification were performed considering the relationship between peak areas vs. standard concentrations at four concentrations (*n* = 4). A linear fitting with a *R* squared (*R*^2^) = 0.99 was obtained.

The FT-IR spectra of CA and SP were recorded by using an ALPHA FTIR spectrometer (Bruker Optics, Ettlingen, Germany) equipped with an ATR (attenuated total reflectance) sampling device containing diamond crystal. The absorbance spectra were recorded between 4000 and 400 cm^–1^, at a spectral resolution of 4 cm^–1^, with 64 scans co-added and averaged. A background spectrum of air was recorded under the same instrumental conditions before each series of measurements. Spectra were processed with the Grams/386 spectroscopic software (version 6.00, Galactic Industries Corporation, Salem, NH).

Due to a very high fluorescence background in the samples, it was not possible to register FT-Raman spectra. Therefore, the SERS effect of silver colloidal solution was employed. SERS spectra of CA and SP were collected on a Renishaw Raman InVia model spectrometer equipped with a Leica microscope electrically cooled CCD camera. Samples were excited by using the 532 nm laser line provided by a frequency−doubled Nd:YAG laser and a power of 2.5 mW at the sample. The spectral resolution was 4 cm^–1^. SERS spectra were recorded with a total acquisition time of 30 s for each SERS spectrum and consisted of four scans. The silver colloid employed to register SERS spectra was prepared by the method of Leopold and Lendl (2003). Briefly, 10 mL of 0.01 M AgNO_3_ solution was added dropwise to 90 mL of a 16 mM solution of hydroxylamine hydrochloride containing 3.33 mM sodium hydroxide. The pH value of the peptide/Ag NPs colloidal mixtures was about 6.5 and the final concentration of products in solution was about 0.01 mM.

### Determination of Hormone (IAA and IPA) Contents and Hormone-Like Activity of Protein Hydrolysates

The content of indole-3-acetic acid (IAA) and isopentenyladenosine (IPA) hormones in the two products was quantified by using the enzyme linked immune-sorbent assay (ELISA) (Sigma, St. Louis, MO, United States), as previously described in Ertani et al. (2013a).

The IAA-like activity was estimated by measuring the reduction of watercress (*Lepidium sativum* L.) roots after the treatment with either IAA or the products, while the gibberellin-like (GA-like) activity was determined by evaluating the increase in the epicotyls length of lettuce (*Lactuca sativa* L.) after application of GA or extracts dilutions (Audus, 1972). The values obtained were the means of 20 samples and five replications.

### Plant Material and Growth Conditions

Seeds of *Zea mays* L. (var. DK C6286, DeKalb, Italy) were soaked in distilled water overnight and then surface-sterilized using 5% (v/v) sodium hypochlorite for 10 min, while shaking. Seeds were left to germinate for 60 h in the dark, at 25°C, on a filter paper wetted with 1 mM CaSO_4_. Germinated seedlings were transplanted into 3 L pots containing an aerated complete culture solution, at a density of 24 plants per pot. The nutrient solution was renewed every 48 h and had the following composition (μM): KH_2_PO_4_ (40), Ca(NO_3_)_2_ (200), KNO_3_ (200), MgSO_4_ (200), FeNaEDTA (10), H_3_BO_3_ (4.6), CuCl_2_ (0.036), MnCl_2_ (0.9), ZnCl_2_ (0.09), NaMoO_4_ (0.01). Plants were cultivated inside a growth chamber, under a 14 h light/10 h dark cycle, air temperature of 27°C/21°C, relative humidity of 70/85%, and photon flux density of 280 mol/m^2^/s. When plants were 12-day-old, they were treated with 0.1 mL L^–1^ or 1 mL L^–1^ (corresponding to 0.1 mg C L^–1^ or 1 mg C L^–1^, respectively) of either CA or SP for 48 h. Untreated plants served as control.

At the 14th day of growth since the transplant, plants were randomly harvested from three pots per treatment, carefully washed and dried with blotting paper. A sub-sample of plant material was immediately frozen with liquid nitrogen and kept at –80°C for further physiological analyses. For plant weight measurement, thirty plants per treatment were used (ten per pot). Roots and leaves of each plants were collected separately, and then weighed.

Root samples were further freeze-dried and analyzed via SERS as described above using a Renishaw Raman InVia model spectrometer equipped with a Leica microscope electrically cooled CCD camera.

### Quantification of Chlorophyll Pigment and Protein Contents in Plants

For chlorophyll content determination, fresh foliar tissue (300 mg) was ground in liquid nitrogen and extracted with 15 mL ethanol (96% v/v). The samples were crushed in liquid nitrogen and kept in the dark for 2 days at 4°C for the extraction. The extracts were filtered and then analyzed spectrophotometrically (UV/VIS Lambda 1; PerkinElmer, Norwalk, CT) at λ = 665 nm for chlorophyll a (Chla) and 649 nm for chlorophyll b (Chlb). The concentration of Chla and Chlb in each sample was calculated using the Wellburn and Lichtenthaler (1984) formula and expressed in mg of pigment per g^–1^ of leaf fresh weight. Two measurements were performed for each plant, using six plants per treatment.

The concentration of soluble proteins was analyzed in leaves and roots of maize plants according to Bradford (1976) using a UV/VIS spectrophotometer (Lambda 1, Perkin-Elmer, Monza, Italy) at λ = 595 nm. Concentrations were expressed as mg of protein g^–1^ fresh weight.

### Determination of Plant Elemental Composition and Total Phenols

Quantification of elements in leaves was obtained after acid digestion by using a microwave (Milestone Ethos model 1600, Milestone, Shelton, CT). Analytical-grade reagents provided by Merck (Merck, Darmstadt, Germany) were used to prepare all solutions. Water was purified using a Milli-Q system (18.2 MΩ cm, Millipore, Bedford, MA). The digestions were carried out as described in Ertani et al. (2016) inside closed Teflon vessels of 120 mL volume using approximately 500 mg dry leaf material and 10 mL of 30% (v/v) HCl. After digestion, the resulting solution was transferred and diluted with 10 mL ultrapure water. Elements were measured via Inductively Coupled Plasma Atomic Emission Spectroscopy (Spectrum CirosCCD, Kleve, Germany).

Total phenols (TP) were determined through the Folin-Ciocalteau (FC) assay using gallic acid as calibration standard (Arnaldos et al., 2001). The FC assay was performed by adding 200 μL of plant extract to 1 mL of Folin-Ciocalteau reagent. Sodium carbonate (800 μL, 20% w/v) was added to the mixture 5 min after the addition of the FC reagent. The mixture was then vortexed for 20 to 30 s. This was recorded as time zero. After 2 h at room temperature, the absorbance of the colored reaction product was measured at λ = 765 nm. The total phenols concentration in the extracts was calculated from a standard calibration curve obtained with different concentrations of gallic acid, ranging from 0 to 600 mg mL^–1^. Results were expressed as milligrams of gallic acid equivalent per kilogram of fresh weight. All measurements were performed using a Shimadzu UV-1800 spectrophotometer (Shimadzu Corp., Columbia, MD, United States).

### Esterase and Peroxidase Enzyme Activities

Esterase activity was performed according to Junge and Klees (1984), while peroxidase activity was measured according to Putter (1974). Leaves and roots (1 g) were homogenized (1:10, w/v) in liquid N_2_ with 0.1 M phosphate buffer (pH 7.0). The extracts were further centrifuged at 15,000 *g* for 15 min at 2°C and the supernatants were collected and used as the enzyme source. The esterase assay was performed by adding 1 mL di α-naphthyl acetate to extracts, and following the release of a free naphthol compound for 20 min at 37°C and λ = 600 nm. The peroxidase assay was carried out by mixing 50 μL of guaiacol, 3%H_2_O_2_, enzyme extract and phosphate buffer to a final volume of 3 mL, and measuring H_2_O_2_ consumption over a 3 min period at λ = 436 nm. Values were expressed as percentages of the control (=0.33 Abs^*^ min^–1^ mg^–1^ fresh weight for leaves, and 0.038 min^–1^ mg^–1^ fresh weight for roots).

### Statistical Analysis

For all determinations, the analysis of variance (ANOVA) was performed using the SPSS software version 19.0 (SPSS Inc., 1999), and was followed by pair-wise *post hoc* analyses (Student–Newman–Keuls test) to determine which means differed significantly at *p* < 0.05 (±SD). The number of biological replicates varied depending on the analysis performed and is indicated in the figures’ and tables’ legends (Sokal and Rohlf, 1969).

## Results

### Chemical Characterization of PHs

The two protein hydrolysates significantly differed in the content of total C (TC) and N (TN), with values significantly higher for CA (Table 1). Specifically, CA contained 1.4-fold and 2-fold more TN and TC, respectively, than SP. With respect to the sulfur content, no significant differences were observed between CA and SP, while the content of organic nitrogen (NO) was weakly higher in SP. The dry matter accounted for 48.59% in CA and 27.04% in SP (Table 1). The content of total phenols (TP) was higher (∼1.45-fold) in CA compared to SP, was well as the amount of individual phenolic acids (chlorogenic acid, caffeic acid, *p*-cumaric acid, and ferulic acid) (Table 1). The content of the reducing sugars glucose and fructose was about 14.4 and 46.2-fold higher, respectively, in CA than in SP. We identified 17 free amino acids in the two products, 10 of which were more abundant in CA, while 4 were occurring at similar concentration. Interestingly, the content of arginine (Arg) was very high in CA (about 14-fold more abundant than in SP) (Table 1).

**TABLE 1:** Content of total carbon (TC), nitrogen (TN), total phenols (TP), dry matter, organic nitrogen (NO), reducing sugars (glucose and fructose), amino acids and individual phenolic acids in the hydrolysates from *Cicer arietinum* (CA) and *Spirulina platensis* (SP).

**Parameters**	**Unit**	**CA**	**SP**
TC	% w/w	23.6 ± 3.24	11.6 ± 1.13
TN	% w/w	2.7 ± 0.12	1.9 ± 0.03
NO	% w/w	5.11 ± 12.18	6.44 ± 12.18
TP	μM	420.0 ± 12.18	610.0 ± 16.31
Dry matter	% w/w	48.59 ± 2.05	27.04 ± 1.13

Glucose	% w/w	3.45 ± 0.57	0.24 ± 1.32
Fructose	% w/w	3.08 ± 0.68	0.07 ± 0.00

Aspartate	% w/w	0.085 ± 0.004	0.029 ± 0.002
Glutamate	% w/w	0.185 ± 0.007	0.120 ± 0.005
Serine	% w/w	0.034 ± 0.002	0.030 ± 0.002
Histidine	% w/w	0.008 ± 0.001	0.005 ± 0.000
Glycine	% w/w	0.033 ± 0.003	0.010 ± 0.001
Threonine	% w/w	0.046 ± 0.003	0.017 ± 0.002
Arginine	% w/w	0.223 ± 0.007	0.009 ± 0.000
Alanine	% w/w	0.120 ± 0.005	0.052 ± 0.004
Tyrosine	% w/w	0.081 ± 0.004	0.021 ± 0.001
Cystine	% w/w	0.011 ± 0.002	0.004 ± 0.000
Valine	% w/w	0.003 ± 0.000	0.001 ± 0.000
Methionine	% w/w	0.123 ± 0.004	0.048 ± 0.003
Phenylalanine	% w/w	0.067 ± 0.003	0.038 ± 0.003
Isoleucine	% w/w	0.063 ± 0.004	0.046 ± 0.002
Leucine	% w/w	0.142 ± 0.003	0.069 ± 0.003
Lysine	% w/w	0.122 ± 0.004	0.041 ± 0.002
Proline	% w/w	0.179 ± 0.004	0.117 ± 0.005

Chlorogenic acid	mg kg^–1^	2.080 ± 0.085	0.682 ± 0.010
Caffeic acid	mg kg^–1^	0.231 ± 0.010	n.d.
*p*-cumaric acid	mg kg^–1^	0.544 ± 0.013	n.d.
Ferulic acid	mg kg^–1^	1.612 ± 0.043	n.d.

*Data represent the means of 3 measurements per treatment (±SD). Data are expressed on a dry matter basis. Analyses were performed on three aliquots of each product. n.d. = not determined.*

### Hormone-Like Activity and Hormone Content in the PHs

In order to establish a possible relationship between the effects exerted by the two hydrolysates on plants and their content in signaling molecules like hormones, the content of IAA and IPA was determined (Table 2). CA and SP contained similar amounts of IAA, but IPA content was ∼5.7-fold higher in CA. The Audus test revealed the presence of both IAA and GA-like activities in the two protein hydrolysates (Table 2). However, while no differences in GA-like activity were evident between CA and SP, the IAA-like activity was ∼3.6-fold more pronounced in CA.

**TABLE 2:** Content of IAA and IPA and hormone-like activity measured in the two hydrolysates obtained from chickpea (*Cicer arietinum* L., CA) and *Spirulina platensis* (SP).

**Protein-hydrolysate**	**IAA**	**IPA**	**IAA-like**	**GA-like**

	**nMol**	**nMol**	**ppm IAA^&^**	**ppm GA^&^**
CA	12.27 ± 3.13	275 ± 7.34	1.07 ± 0.05	0.10 ± 0.03
SP	11.82 ± 1.23	48 ± 5.02	0.30 ± 0.01	0.10 ± 0.01

*Data represent the means of 3 measurements per treatment (±SD). ^&^ IAA- and GA-like activities are expressed as ppm IAA and GA. which correspond to the concentration of either indoleacetic acid or gibberellic acid of equivalent activity as 1 mg C L^–1^.*

### FT-IR and SERS Spectroscopies of the PHs

**FIGURE 1:**
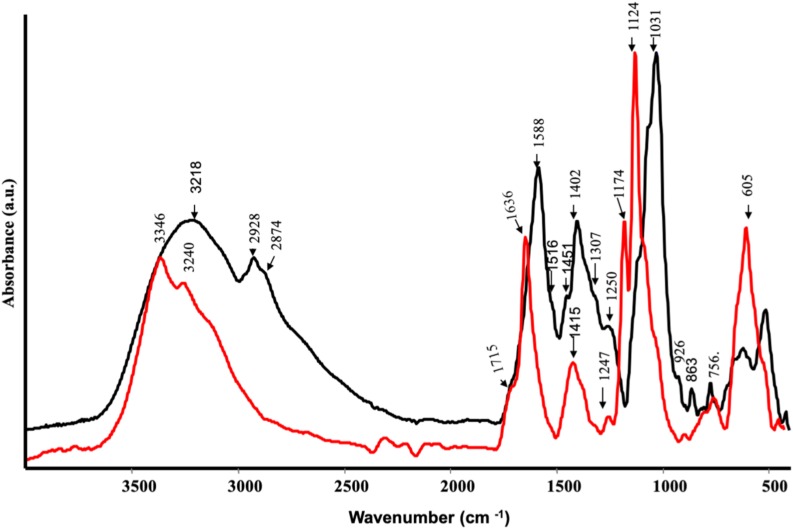
ATR-FTIR spectra of protein hydrolysates from *Spirulina platensis* (red) and *Cicer arientinum* (black).

Some functional groups have been well identified in the CA extract by FT-IR (Figure 1). A broad band at 3218 cm^–1^ was due to OH stretching, a shoulder at 2928 cm^–1^ and at 2874 cm^–1^ were assigned to –CH_2_ asymmetric and –CH_2_ symmetric stretching, respectively. In addition, a strong band at 1588 cm^–1^ was likely due to –COO^–^ asymmetric stretching of carbohydrate group, a medium band intensity at 1402 cm^–1^ owing to –CH bending and symmetric stretching of – COO^–^ (Fan et al., 2012). A weak shoulder at 1516 cm^–1^ corresponded to aromatic C=C stretching and the band at 1250 cm^–1^ was attributed to C–OH stretching in acids or phenols. The strong peaks at 1031 cm^–1^ was probably ascribed to C–O of C–O–C or skeletal mode vibrations of α-1,4 glycosidic linkage of C–O–C in starch (Fan et al., 2012). The spectrum of SP (Figure 1) was characterized by a broad band at 3346 and 3240 cm^–1^ assigned to O–H stretching, while a slight shoulder at 3084 cm^–1^ could be assigned to C=C–H stretching vibration. The presence of a slight shoulder at 1715 cm^–1^ and a weak band at 1247 cm^–1^ indicated the C=O and C–OH stretching vibration of acidic groups, respectively. The bands at 1636 and 1415 cm^–1^ were attributed to the asymmetric and symmetric stretching of carboxylate groups, respectively, while the bands in the 1174 to 892 cm^–1^ spectral range were typical of the C–OH and C–O–C vibration of polysaccharides (Fan et al., 2012).

**FIGURE 2:**
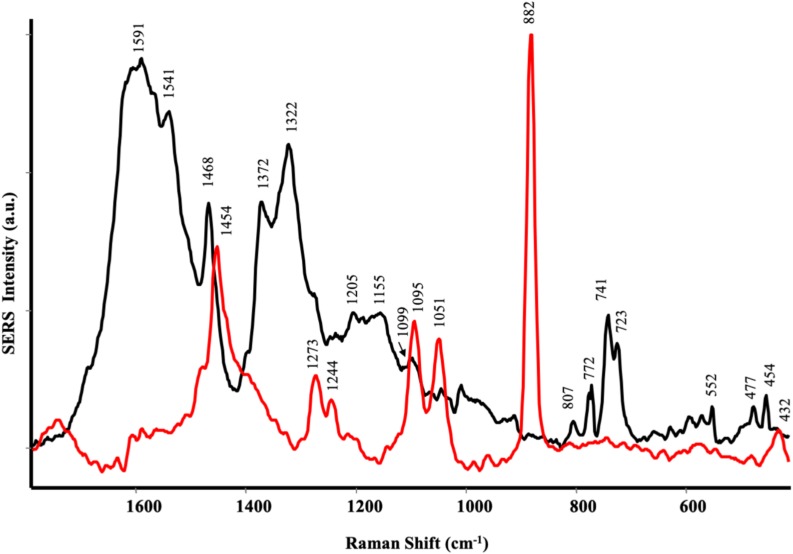
ATR spectra of protein hydrolysates from *Spirulina platensis* (red) and *Cicer arientinum* (black).

Surface Enhanced Raman Scattering spectra (Figure 2) confirmed the findings of IR spectroscopy. As a matter of fact, CA showed many peaks attributable to phenolic substances: the bands at 1591 and 1468 cm^–1^ were diagnostic stretching vibration of highly substituted aromatic ring of phenolic compounds, together with the bands at 1322 and 1205 cm^–1^, typical of phenolic substituents (C–OH stretching vibration) (Francioso et al., 2002). Other typical bands of phenols appeared at 1155 and 1099 cm^–1^ (mixed vibrations arising from C–O, C–C, and C–H bonds) and at 807, 772, 723, 552, 477, and 454 cm^–1^ (–OCH_3_ and skeletal vibrations) (Sanchez-Cortes and Garcìa-Ramos, 2000) The presence of the band at 1372 cm^–1^ was attributed to the symmetric stretching of COO^–^, indicating that these polyphenols contained the carboxylate group (Francioso et al., 2002). The spectrum of SP (Figure 2) was dominated by alginate vibrations (Campos-Vallette et al., 2010): CH_2_ stretching vibrations at 1454 cm^–1^, ring C–H bending at 1273 cm^–1^, C–O stretching at 1244 cm^–1^, carbohydrate C–O stretching motions at 1095, 1051 and the band at 882 cm^–1^, attributed to the anomeric carbon atom in saccharides. Finally, the skeletal ring deformation appearing at 432 cm^–1^.

### SERS of Plant Tissues

**FIGURE 3:**
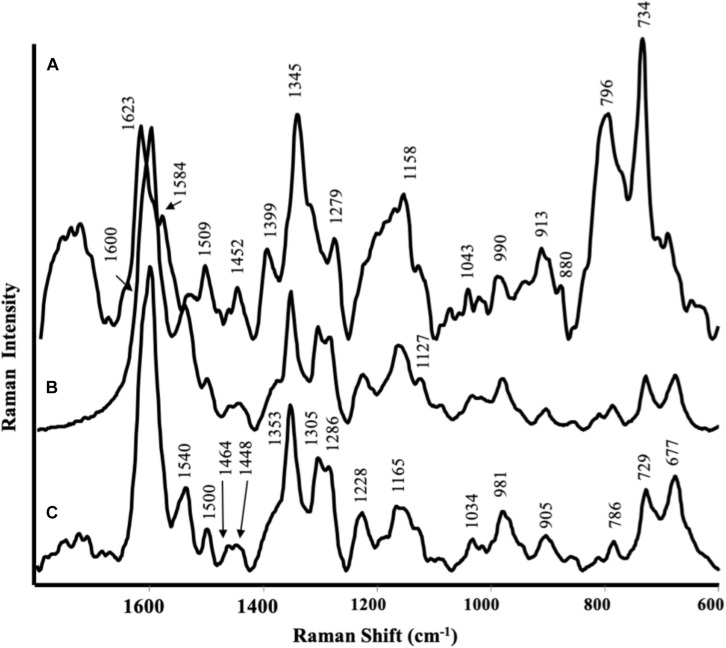
Surface-enhanced Raman scattering (SERS) spectra of maize roots treated with protein hydrolyzed from **(A)** *Spirulina platensis*; **(B)** *Cicer arietinum* L., and **(C)** untreated.

Surface Enhanced Raman Scattering spectra of the root tissues were dominated by phenolic substances of different nature that strongly interacted with Ag nanoparticles (Figures 3A,B). The SERS spectrum of roots treated with CA extracts (Figure 3B) was very similar to that recorded for control roots (Figure 3C): the bands in the 1600–1280 cm^–1^ spectral region (i.e., at 1600, 1540, 1500, 1464, 1353, 1305, and 1286 cm^–1^) could be attributed to ring stretching vibration; the bands at 1228 and 1165 cm^–1^ corresponded to mixed vibrations arising from C–O, C–C, and C–H bonds, while bands at 981, 905, 729, and 677 cm^–1^ were typical of C–H and C–C vibrations. All these bands have been previously described by several authors (Sanchez-Cortes and Garcìa-Ramos, 2000; Aguilar-Hernández et al., 2017) in the SERS spectra of phenolic compounds, such as caffeic acid, ferulic acid, catechol, and gallic acid.

In the case of plants treated with the SP product (Figure 3A), the spectrum was different from that referred to control and the main bands observed could be attributed to other phenolic substances, like coumaric or sinapic acid (Aguilar-Hernández et al., 2017): 1623 and 1584 cm^–1^ were attributed to phenyl ring deformations; 1345 cm^–1^ to COO^–^ stretching; 1279 cm^–1^ to C–O stretching, 1558 cm^–1^ to C–H bending. The very intense bands in the 800–700 cm^–1^ spectral region, together with the band at 1399 cm^–1^, could be assigned to highly substituted aromatic compounds analogous to the alkaloid berberine (Leona and Lombardi, 2007): the last one were likely due to in plane ring vibrations, while the bands at 796 and 734 cm^–1^ to out of plane ring deformations. The less intense bands appearing at 1452, 1279, and 880 cm^–1^, present also in the SERS spectra of the SP hydrolysate, could be attributed to substances like alginate (Campos-Vallette). Unfortunately, no SERS spectra were obtained by leaves due to the strong florescence from the samples.

### Effect of CA and SP on Leaf and Root Biomass Production

**FIGURE 4:**
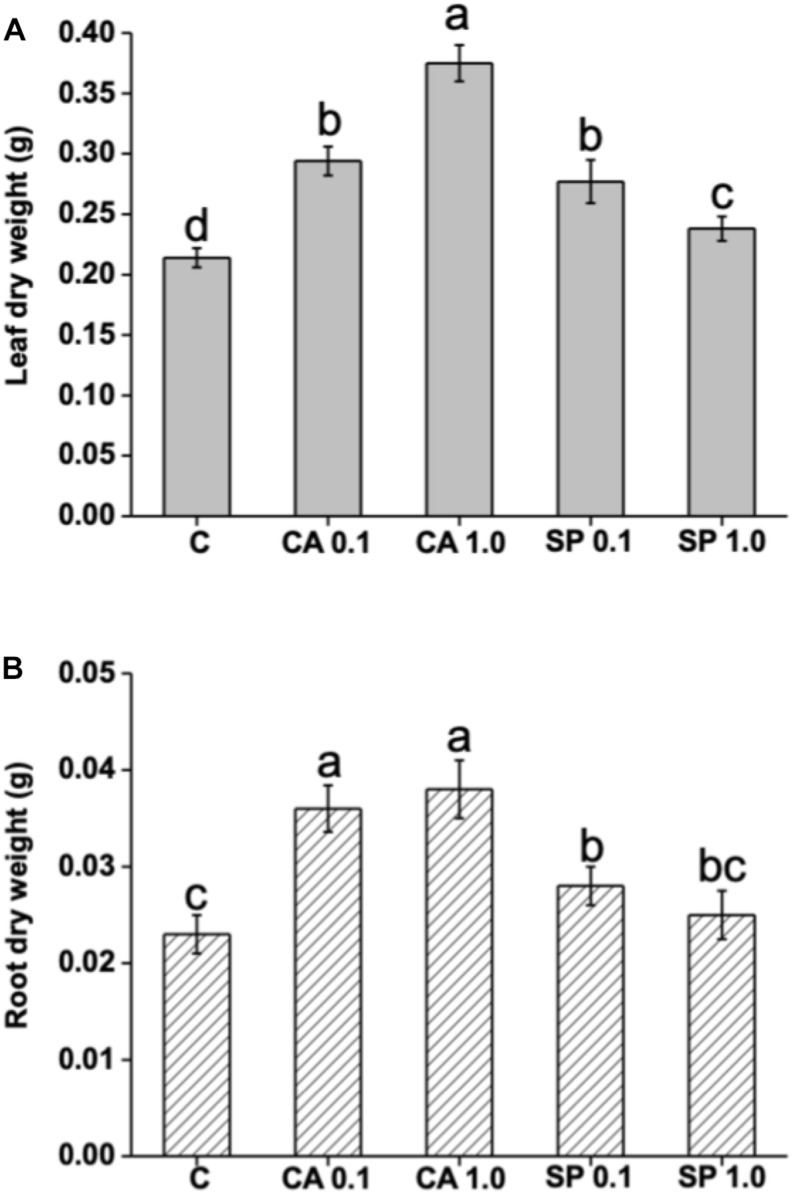
Effect of two protein-hydrolysates (CA and SP), supplied for 48 h at two dosages (0.1 and 1 mL L^–1^) on leaf and root dry weight of *Z. mays* plants. CA derived from chickpea (*Cicer arietinum* L.) and SP from *Spirulina platensis* (SP). Different letters above bars indicate significant differences (*p* < 0.05) among leaves **(A)** and roots **(B)**. Data are the means of 30 values, each from three independent experiments (±SD).

The protein hydrolysates CA and SP were furnished to 2 week-old maize plants for 48 h at 0.1 and 1 mL L^–1^, and their effects on plant growth in terms of dry biomass production were evaluated. Results indicated that both CA and SP caused significant increases in leaf and root dry weight (Figures 4A,B). However, CA was more efficient than SP to stimulate plant growth. The most prominent effects were observed in leaves of plants treated with either SP at the low or CA at the high dosage, with values 29 and 75% higher, respectively, than the corresponding controls. With respect to roots, a similar trend of dry biomass production in response to CA and SP treatments was observed as in leaves.

### Effect of CA and SP on Chlorophyll, Protein and Total Phenol Accumulation

To evaluate the effect of CA and SP hydrolysates on plant N metabolism and productivity, the concentrations of chlorophylls, proteins and total phenols (TP) were determined (Table 3). Both CA and SP appreciably increased the protein concentration in leaves and roots of maize plants, especially when supplied at the high (1 mL L^–1^) dosage. When plants were treated with CA at such dosage, protein accumulation was 33 and 45% higher in leaves and roots, respectively, compared to corresponding controls. Total phenol accumulation was also more increased by high CA and SP dosages, but in this case to similar levels. Chlorophyll content was, however, more abundant when plants were furnished with the low (0.1 mL L^–1^) dosage of either CA or SP.

**TABLE 3:** Content of proteins in leaves and roots, chlorophyll and total phenols (Phe) in leaves of maize plants treated for 48 h with two dosages (0.1 and 1 mL L^–1^) the protein-hydrolysates from either chickpea (*Cicer arietinum* L., CA) or *Spirulina platensis* (SP).

**Treatments**		**Protein**		**Chlorophyll**	**TP**

	**mL L^–1^**	**mg g^–1^ f.wt.**		**μg Chl g f.wt.**	**mg gallic acid g^–1^ f.wt.**

		**Leaves**	**Roots**	**Leaves**	
Control	-	3.10 ± 0.18c	1.11 ± 0.11b	8.77 ± 1.32d	1.22 ± 0.04b
CA	0.1	3.85 ± 0.33a	1.55 ± 0.10a	14.46 ± 2.11b	1.38 ± 0.12a
CA	1.0	4.12 ± 0.31a	1.61 ± 0.12a	10.91 ± 1.20cd	1.44 ± 0.10a
SP	0.1	3.44 ± 0.13bc	1.46 ± 0.13a	20.24 ± 2.14a	1.35 ± 0.05a
SP	1.0	3.72 ± 0.21ab	1.54 ± 0.10a	11.36 ± 3.27c	1.43 ± 0.04a

*Different letters along columns indicate significant differences among treatments (p < 0.05, n = 3, ±SD).*

### Effect of CA and SP on Plant Mineral Nutrients

The concentrations of a number mineral nutrients (Ca, K, Mg, Cu, Zn) were determined to assess whether CA and SP could stimulate plant nutrition via promotion of nutrient uptake and accumulation. In general, both hydrolysates substantially increased the foliar and root concentrations of macro- and micro-elements compared to the control plants (Table 4). Maximum leaf macroelement accumulation was evident in plants treated with CA at 1 mL L^–1^. Specifically, in these plants the contents of Ca, K and Mg were 59, 136, and 37% higher, respectively, than in the corresponding controls. In roots, the most remarkable increments were observed for Mg, which was ∼2.3-fold more accumulated when plants were provided with CA.

**TABLE 4:** Content of macro- (Ca, K, and Mg) and micro- (Zn, Cu) elements in leaves and roots of maize plants treated for 48 h with two dosages (0.1 and 1 mL L^–1^) the protein-hydrolysates from either chickpea (*Cicer arietinum* L., CA) or *Spirulina platensis* (SP).

**Treatments**		**Ca**	**K**	**Mg**	**Zn**	**Cu**

	**mL L^–1^**		**g kg**^–1^		**mg kg**^–1^	

		**Leaves**						
Control	–	7.24 ± 0.87b	22.53 ± 3.32c	6.38 ± 0.34c	93.15 ± 12.38b	21.62 ± 6.22b
CA	0.1	13.21 ± 1.08a	35.93 ± 1.38b	8.46 ± 1.03a	122.12 ± 12.67a	38.77 ± 8.33a
CA	1.0	13.84 ± 1.22a	53.12 ± 2.35a	8.74 ± 1.12a	106.22 ± 11.09ab	34.31 ± 13.38a
SP	0.1	7.31 ± 0.21b	37.54 ± 1.38b	7.84 ± 0.67b	134.13 ± 13.27a	32.53 ± 11.03a
SP	1.0	12.87 ± 1.13a	34.24 ± 2.49b	8.13 ± 1.15a	128.13 ± 11.04a	37.81 ± 9.21a

**Roots**						

C	–	6.39 ± 1.17c	12.87 ± 1.11b	7.53 ± 0.34c	278.54 ± 10.11	21.62 ± 6.22b
CA	0.1	9.45 ± 1.17a	15.87 ± 1.22a	17.34 ± 2.30a	297.47 ± 13.67a	38.77 ± 8.33a
CA	1.0	11.33 ± 1.12a	15.53 ± 1.13a	16.36 ± 2.21ab	402.43 ± 11.09ab	34.31 ± 13.38a
SP	0.1	8.57 ± 1.33b	16.77 ± 2.64a	12.48 ± 2.24b	297.47 ± 13.67	32.53 ± 11.03a
SP	1.0	8.36 ± 1.10b	15.44 ± 1.12a	15.36 ± 2.14ab	402.43 ± 23.17	37.81 ± 9.21a

*Different letters along columns indicate significant differences among treatments (p < 0.05, n = 3, ±SD).*

### Effect of CA and SP on Esterase and Peroxidase Enzyme Activities

**FIGURE 5:**
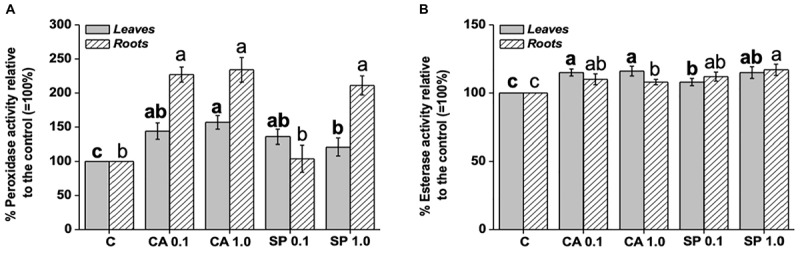
Leaf and root activity of peroxidase **(A)** and esterase **(B)** in maize plants treated for 48 h with two dosages (0.1 and 1 mL L^–1^) of the protein hydrolysates from either chickpea (*Cicer arietinum* L., CA) or *Spirulina platensis* (SP). Different letters above bars indicate significant differences (*p* < 0.05) among leaves (bold) and roots (unbolded). Data are the means of 5 values each from three independent experiments (±SD).

Application of CA and SP protein hydrolysates to maize plants caused a significant increase in the activity of peroxidase (Figure 5A). The effect was more pronounced when plants were supplied with CA, regardless of the applied dosage. In addition, peroxidase activity was more enhanced in roots than in leaves by both CA dosages, with values 130% higher than the relative controls. It was also stimulated by low SP dosage, with values 111% higher than the corresponding control. With respect to the esterase enzyme, the supply of CA and SP determined weaker increments in its activity compared to peroxidase (Figure 5B). CA at both dosages in particular, stimulated the esterase activity by 15–16% and 8–10% in leaves and roots, respectively, compared to the corresponding controls. On the other side, SP induced a dosage-dependent increase of peroxidase activity, both in leaves and roots.

## Discussion

The use of biostimulants in agriculture represents an intriguing sustainable strategy for improving crop yield. Research in this field is focused toward the generation of new types of products and the evaluation of their effectiveness in improving crop productivity (Rouphael and Colla, 2018). Therefore, in the current study, two protein hydrolysates provided by ILSA Spa and obtained from chickpea (*C. arietinum L*.) and a microalga (*S. platensis)* have been characterized and further applied to maize plants in order to ascertain their effects on some physiological traits related to productivity. The use of chickpea and *S. platensis* as matrices for the production of the two protein hydrolysates was justified by their contents in valuable phytochemicals. Among legumes, chickpea represents a valuable source of dietary proteins and a main constituent of daily diet in several countries (Jukanti et al., 2012). A number of studies have highlighted the abundance of polyphenols in this species, especially isoflavones, either as aglycones or as different types of glycoside conjugates (Aguilera et al., 2009). The isoflavones daidzein and genistein (phytoestrogens), whose chickpea is notoriously rich, are also used by plants as signal carriers, as well as for defense responses to pathogenic attacks (Jung et al., 1999). The microalga *S. platensis* is a very good source of carbohydrates, as revealed by the presence of carboxylate and glycosidic linkage of C–O–C in both spectra, but it is also abundant in proteins and other phytochemicals. Nevertheless, its composition varies depending on tillage conditions. When compared with extracts obtained from other algae that we previously tested as biostimulants (Ertani et al., 2018), the *S. platensis* protein hydrolysate contained more N and C than *Laminaria* and most *A. nodosum* extracts.

The two protein hydrolysates substantially differed in terms of chemical constituents and functional groups. Such differences likely depended on the raw material used as a source for their production. The protein hydrolysate from chickpea in particular, contained higher levels of N, C, total phenolic compounds, amino acids, soluble sugars, IAA and IPA. CA also displayed higher IAA-like activity. The presence of phenolic substances was also supported by FT-IR and SERS spectra and analysis of individual phenolic acids. In more details, SERS spectra of CA showed typical bands of polyphenols highly substituted and phenolic acids.

Differences in chemical and hormone composition between CA and SP were likely responsible for their effects on maize plant growth and accumulation of N compounds (proteins, chlorophylls and phenolics). Indeed, although both CA and SP positively influenced the maize biomass production, acquisition of nutrient elements and accumulation of polyphenols, these effects were more pronounced when plants were supplied with CA. As mentioned above, CA contained more total phenols than SP, although the analysis of individual phenolic acids revealed higher content of chlorogenic acid, caffeic acid, *p*-cumaric acid and ferulic acid in SP. It is noteworthy that phenols function as signaling molecules in plants and in suitable amounts elicit positive responses on plant growth, also whether they are applied exogenously as components of biostimulants (Ertani et al., 2011a, 2018). In a previous work, extracts from *Laminaria* and *A. nodosum* were examined for their capacity to promote plant growth in relation to their content in phenols (Ertani et al., 2018). Extracts containing the lowest levels of total phenols (within physiological concentrations) were the least efficient in stimulating root development, even though they exhibited appreciable IAA-like activity. Based on these considerations, higher phenol content measured in CA could have been responsible for greater stimulation of maize plant growth. In addition, CA contained elevate levels of the amino acid Arg, which is a precursor of polyamines, i.e., pivotal compounds required to start the cell division process in plants (Popko et al., 2018), and of the reducing sugars glucose and fructose, whose availability to plants has been reported to affect cell division as well (Ciereszko, 2018). In this respect, total phenols, amino acids and sugars in CA likely acted synergically and in combination with high hormone (IAA and IPA) content and IAA-like activity to stimulate plant growth. The positive effects of auxins and/or substances with auxin-like activity contained in several types of biostimulants on lateral root and hair formation are well established (Schiavon et al., 2010; Spinelli et al., 2010; Ertani et al., 2013b,c, 2018).

The increase in root biomass by CA and SP was probably responsible for higher capacity of nutrient acquisition by plants compared to the untreated controls (Ertani et al., 2018). On the other hand, bioactive molecules (phenols, sugars and amino acids) contained in the two protein hydrolysates may have acted as signaling molecules for the up-regulation of genes coding for nutrient transporters, and thus in turn may have lead to the enhancement of plant biomass production (Billard et al., 2014; Ertani et al., 2017b). It can be also hypothesized that amino acids and sugars in the two protein hydrolysates provided carbon skeletons to be converted into precursors or intermediates of the tricarboxylic acid cycle, therefore contributing to the respiratory metabolism and ATP production for energy-dependent processes, like the transport of nutrients. In previous studies, the effect of a protein hydrolysate obtained from *alfa-alfa* plants was shown to improve elemental composition of *Solanum lycopersicon* plants via up-regulation of several genes coding for transporters of macro- and micro-elements (Ertani et al., 2017b), while extracts from the microalgae *Chlorella vulgaris* and *Scenedesmus quadricauda* have been recently reported to be efficient in up-regulating genes related to nutrient acquisition (Billard et al., 2014). Nitrogen assimilation was enhanced by CA and SP, as confirmed by increased production of chlorophylls, proteins and phenols. In support of this, previous works reported similar effects due to protein hydrolysate application (Schiavon et al., 2008; Ertani et al., 2009).

Peroxidase enzymes, in addition to be involved in the plant responses to abiotic and biotic stresses via scavenging of Reactive Oxygen Species (ROS), are recognized together with esterase enzymes as predictive indicators of plant growth and cellular differentiation processes (Balen et al., 2003). Previously, humic substances and seaweed extracts have been shown to stimulate the activity of such enzymes when plants were grown under no stress conditions (Nardi et al., 2000; Ertani et al., 2018). In our study the most pronounced increments in activity were observed for peroxidase, in plants treated with CA. Peroxidase is also implied in the phenylpropanoid pathway that leads to the biosynthesis of lignin. Therefore, its strong increase in activity observed in CA-treated plants could be at least partially related to higher accumulation of phenols, including lignin precursors, measured in the same plants.

In conclusion, the two hydrolysates tested in the current research showed positive effects on maize plants, as they stimulated plant growth, acquisition of nutrient elements and higher synthesis of phytochemicals including proteins, phenolics and chlorophylls. CA appeared the most efficient. It is possible that signal transduction routes elicited by higher content of phenols, sugars and certain amino acids in CA have interacted with other pathways in the plants creating a complex communication and signaling network involved in the plant growth processes. Spectroscopic analyses and chemical characterization of PHs were useful tools in supporting experiments with these products aimed at evaluating their stimulatory effects on plants.

## Data Availability

The raw data supporting the conclusions of this manuscript will be made available by the authors, without undue reservation, to any qualified researcher.

## Author Contributions

OF, MDF, and SS-C performed the spectroscopic analysis and wrote the relative part in the manuscript. AE wrote the manuscript and performed the physiological analyses, bioassays and chemical analyses of the products. MS wrote the manuscript. SN designed the study. All authors critically reviewed the manuscript.

## Conflict of Interest Statement

The authors declare that the research was conducted in the absence of any commercial or financial relationships that could be construed as a potential conflict of interest.
